# Recent advances in understanding and managing adenomyosis

**DOI:** 10.12688/f1000research.17242.1

**Published:** 2019-03-13

**Authors:** Silvia Vannuccini, Felice Petraglia

**Affiliations:** 1 Department of Neuroscience, Psychology, Pharmacology and Child Health (NEUROFARBA), University of Florence, Florence, Largo Brambilla 3, 50134, Italy; 2 Department of Molecular and Developmental Medicine, University of Siena, Siena, viale Mario Bracci 16, 53100, Italy; 3 Obstetrics and Gynecology, Department of Maternity and Child Health, University Hospital Florence, Careggi University Hospital, Florence, Largo Brambilla 3, 50134, Italy; 4 Department of Experimental, Clinical and Biomedical Sciences, University of Florence, Florence, Largo Brambilla 3, 50134, Italy

**Keywords:** abnormal uterine bleeding, adenomyosis, endometriosis, histology, hormonal treatment, infertility, magnetic resonance imaging (MRI), medical treatment, pelvic pain, pregnancy outcome, transvaginal ultrasound sonography (TVUS), uterine fibroids.

## Abstract

Adenomyosis is a benign uterine disorder in which endometrial glands and stroma are pathologically demonstrated in the uterine myometrium and it is considered a specific entity in the PALM-COEIN FIGO (polyp; adenomyosis; leiomyoma; malignancy and hyperplasia; coagulopathy; ovulatory dysfunction; endometrial; iatrogenic; and not yet classified – International Federation of Gynecology and Obstetrics) classification of causes of abnormal uterine bleeding (AUB). Although it has always been considered the classic condition of multiparous women over 40 years old who have pain and heavy menstrual bleeding, diagnosed at hysterectomy, the epidemiological scenario has completely changed. Adenomyosis is increasingly identified in young women with pain, AUB, infertility, or no symptoms by using imaging techniques such as transvaginal ultrasound and magnetic resonance. However, there is no agreement on the definition and classification of adenomyotic lesions from both the histopathology and the imaging point of view, and the diagnosis remains difficult and unclear. A uniform and shared reporting system needs to be implemented in order to improve our understanding on imaging features, their relationship with pathogenic theories, and their importance in terms of clinical symptoms and response to treatment. In fact, adenomyosis pathogenesis remains elusive and not a single theory can explain all of the different phenotypes of the disease. Furthermore, adenomyosis often coexists with other gynecological conditions, such as endometriosis and uterine fibroids, increasing the heterogeneity of available data. Treatment requires a lifelong management plan as the disease has a negative impact on quality of life in terms of menstrual symptoms, fertility, and pregnancy outcome and has a high risk of miscarriage and obstetric complications.

## Introduction

Adenomyosis is a benign uterine disorder in which endometrial glands and stroma are pathologically demonstrated in the myometrium ^1^. Women affected by adenomyosis may present with abnormal uterine bleeding (AUB), dysmenorrhea, dyspareunia, or infertility but one third of them are asymptomatic ^2^. For many years, adenomyosis has remained a histopathological diagnosis made after hysterectomy in perimenopausal women with heavy menstrual bleeding (HMB) or pelvic pain ^3^. Over the last decade, adenomyosis has also become a condition identified in young fertile-age women ^4^ thanks to the recent advancements in imaging techniques, even though a shared definition and classification are still lacking ^5^. Despite the improvement of diagnostic tools, the awareness of the condition is still poor. Furthermore, in some patients, adenomyosis coexists with other gynecological conditions, such as endometriosis and uterine fibroids ^6^. The physio-pathological mechanisms, involving sex steroid hormone aberrations, inflammation, fibrosis, and neuroangiogenesis, are not fully understood ^7^. Regarding the current management of adenomyosis, there are no international guidelines to follow for surgical or medical treatment of adenomyosis ^8^ and this will be of utmost importance in the future as the disease requires a lifelong management plan, including pain and bleeding control, fertility preservation, and pregnancy outcome.

## Pathogenesis

The pathogenic mechanisms involved in adenomyosis need to be fully elucidated, but in the last decade an increasing number of studies have shown that sex steroid hormone receptors, inflammatory molecules, extracellular matrix enzymes, growth factors, and neuroangiogenic factors play a major role ^7, 9, 10^.

According to the most common theory, adenomyosis results from the invagination of basalis endometrium into the myometrium through an altered or interrupted junctional zone (JZ) ^7, 11^, which represents a highly specialized hormone-responsive structure located in the inner third of the myometrium ^12^. Molecular alterations in eutopic endometrium seem to contribute to migration and survival of ectopic endometrial implants beyond the myometrial interface ^13^. Even though alterations in apoptosis, steroid hormone responsiveness, and extracellular matrix pathways have been found in both adenomyotic lesions and eutopic endometrium, the underlying mechanisms need to be further evaluated. In addition, the role of tissue injury and repair (TIAR) as the primary mechanism for myometrial invasion has been hypothesized ^6^. Chronic peristaltic myometrial contractions may induce continuous microtrauma to the JZ, causing inflammation which in turn promotes local increased estrogen production, inducing a vicious cycle. A positive feedback mechanism is generated, and chronic hyperperistalsis in the JZ promotes repeated cycles of autotraumatization ^14^. Thus, the TIAR theory, stressing the importance of tissue damage to the endometrial–myometrial interface, supports the common understanding that adenomyosis is associated with multiparity, previous cesarean section, and prior uterine surgery ^11^.

An alternative pathogenic theory of adenomyosis proposes that the disease arises *de novo* from metaplasia of embryonic or adult stem cell in the myometrium ^15^. Intramyometrial embryonic pluripotent Müllerian remnants may undergo metaplastic changes in the adult uterine wall, leading to the establishment of *de novo* ectopic endometrial tissue within the myometrial wall, as adenomyotic foci. However, the hypothesis of adult endometrial and stromal stem cell differentiation whenever they are deposited in the myometrium after retrograde menstruation should also be taken into account ^11, 16^. Accordingly, Chapron *et al*. described “from outside to inside invasion” theory, hypothesizing the migration of ectopic endometrial cells from posterior endometriosis nodules into the myometrium ^17^. This theory was supported by the high prevalence of posterior focal adenomyosis of the outer myometrium (FOAM) found in patients with deep infiltrating endometriosis (DIE) nodules in the posterior compartment, diagnosed by magnetic resonance imaging (MRI) ^17^. After retrograde menstruation, ectopic endometrial cells may have the potential to infiltrate not only pelvic organs but also the uterine walls. Less clear is the explanation for the 50% association of anterior FOAM shown in bladder DIE ^18^. The intraperitoneal seeding of endometrial cells after menstruation may cause invasion of the vesicouterine pouch, generating both a bladder nodule and anterior FOAM through a trans-serosa invading process. Nevertheless, the pathogenesis of adenomyosis is still unclear and cannot be understood by only a unique theory since the phenotypes are heterogeneous and not clearly defined.

## Epidemiology

Historically, adenomyosis has been identified as a pathological entity from histological reports after hysterectomy. However, currently, only a small group of women undergo non-conservative surgical treatments for adenomyosis; thus, a realistic epidemiological background cannot be established from histopathology. Furthermore, the introduction of new medical compounds and surgical techniques has allowed clinicians to conservatively treat the disease. Thus, the epidemiological scenario has changed, and although the most common risk factor profile ^19^ included age of more than 40 years, multiparity, prior cesarean section, or uterine surgery ^19, 20^, the disease is increasingly diagnosed in young women ^4^, in infertility patients ^21^, or in those with pain or AUB or both ^22^. In regard to AUB, in fact, adenomyosis is considered a specific entity in the PALM-COEIN FIGO (polyp; adenomyosis; leiomyoma; malignancy and hyperplasia; coagulopathy; ovulatory dysfunction; endometrial; iatrogenic; and not yet classified – International Federation of Gynecology and Obstetrics) classification ^23, 24^ and it is strictly associated with HMB ^22^. However, it should also be considered that one third of patients with adenomyosis are asymptomatic ^2^. In women undergoing assisted reproductive technologies (ARTs), the prevalence of adenomyosis is 20% to 25% ^21^, whereas in those with a history of endometriosis, the percentage is widely variable, ranging from 20% to 80% ^25, 26^. Data from ultrasound units show a 20.9% prevalence of sonographic signs of adenomyosis in the general population ^27^, whereas the figures range from 10% to 35% in histological reports after hysterectomy ^28, 29^. However, uniform diagnostic criteria, in both histopathology and imaging, are needed in order to standardize the diagnosis and to have reliable updated figures.

## Definition and diagnosis of adenomyosis

### Histology

The gold standard to make the final diagnosis of adenomyosis has always been considered histological examination of hysterectomy specimens ^23^. So, in 2009, Weiss *et al*. declared that adenomyosis was an incidental finding in women undergoing hysterectomy and not the source of their symptoms ^30^. The condition is commonly described as the presence of endometrial glands and stroma located deep within the myometrium, associated with smooth muscle hyperplasia ^31^. This is due to the invasion of myometrium by basal glands and stroma with destruction of normal myometrium architecture ^32^. However, the frequency of adenomyosis diagnosis can range widely among different pathologists as there are no uniform criteria regarding depth of invasion and number of foci to make a diagnosis, especially when disease is not diffuse. In previous papers, an invasion more than one third thickness of the uterine myometrium has been considered to make a diagnosis, whereas in others a myometrial invasion greater than 4 mm was diagnostic for adenomyosis ^33– 35^. Regarding histological classification, adenomyosis is defined as focal when circumscribed nodular aggregates of endometrial glands and stroma are surrounded by normal myometrium. Conversely, an adenomyoma is identified when the ectopic endometrial tissue within the myometrium is surrounded by hypertrophic myometrium. In diffuse adenomyosis, endometrial glands and stroma are recognized throughout the myometrium.

However, the available criteria do not consider potential different morphological appearance of ectopic cells according to cellular differentiation stage and menstrual cycle or responsiveness to hormonal drugs. In addition, results from histopathological studies are biased by the fact that in those cases all women underwent a non-conservative treatment; thus, this does not represent the entire population of adenomyotic patients or all possible phenotypes.

### Imaging

The development of imaging techniques, such as MRI and transvaginal ultrasonography (TVUS), has allowed clinicians to make a non-invasive diagnosis of adenomyosis in women also undergoing conservative treatments, identifying different phenotypes of the disease. After some attempts of histological classification ^36^, Kishi *et al*. (in 2012) classified adenomyosis in four subtypes according to MRI lesion localization in the inner or outer myometrium: intrinsic, extrinsic, intramural, and indeterminate ^37^. In 2014, Grimbizis *et al*. proposed a clinical histological classification system, identifying diffuse, focal, and cystic adenomyosis ^38^. More recently, Bazot and Daraï proposed three subtypes—internal, external adenomyosis, and adenomyomas—according to MRI features ^39^. However, a shared classification system has not been developed yet as further research is needed in order to better understand the physiopathology of adenomyosis, its onset and progression, and the interpretation of imaging signs according to the pathogenic theories.

***Transvaginal ultrasound sonography*.** TVUS represents the first-line imaging technique to diagnose adenomyosis as it is widely available, relatively inexpensive, and very accurate if performed by expert sonographers. The sensitivity of TVUS to detect adenomyosis ranges from 65% to 81%, and specificity ranges from 65% to 100%. A recent meta-analysis, pooling results from eight studies, showed that two-dimensional TVUS has a sensitivity and specificity of 83.8% and 63.9%, respectively, and that for three-dimensional TVUS, pooled sensitivity and specificity for all combined imaging characteristics are 88.9% and 56.0%, respectively ^40^. Recently, a uniform standardized reporting system of ultrasound findings of adenomyosis was developed by using the Morphological Uterus Sonographic Assessment (MUSA) criteria ^41^. According to those criteria, the typical ultrasound features to consider in order to make a diagnosis of adenomyosis are described as follows: asymmetrical thickening of uterine walls, intramyometrial cysts or hyperechoic islands (or both), fan-shaped shadowing of the myometrium, myometrial echogenic subendometrial lines and buds, translesional vascularity, and irregular or interrupted JZ. However, there is little available evidence linking the ultrasound features with histopathology ^42, 43^, even though the different sonographic characteristics may be explained by the relative proportion of endometrial glandular structures, endometrial stroma, and hypertrophic muscle elements.

The new reporting system of adenomyosis, proposed by Van den Bosch and de Bruijn *et al*., includes the description of disease location (anterior, posterior, left lateral, right lateral, and fundal), classification of the lesions as focal or diffuse, presence or absence of intralesional cysts, myometrial layer involvement (JZ, myometrium, and serosal involvement), disease extent (<25%, 25%–50%, and >50% of uterine volume affected by adenomyosis), and lesion size ^44^. This system would help to standardize the ultrasound description of adenomyosis; however, it has some limitations to be fully implemented in clinical practice. For example, the measurement of uterine volume affected by the disease is difficult to be objectively evaluated. Furthermore, the system needs to be validated and to be correlated with clinical findings and fertility outcomes in further studies. In fact, another major problem is the definition of severity of the disease and the identification of those features that make clinically more severe adenomyosis. Previously, Naftalin *et al*. showed a positive correlation between the number of ultrasound features of adenomyosis and the severity of menstrual pain, but there is no available evidence in which features are relevant from a clinical perspective ^45^. Recently, a scoring system to define the severity of adenomyosis was proposed ^46^; similarly, Tellum *et al*. developed a clinical prediction model to identify the disease by using the most relevant features ^47^. However, those models need to be externally validated and more evidence should support their reliability.

***Magnetic resonance imaging*.** MRI diagnosis of adenomyosis is essentially linked to the thickening of the JZ, but it also includes direct and indirect signs of the presence of endometrial glands within the myometrium and smooth muscle cell hypertrophy ^48^. Typical adenomyosis appears as an ill-demarcated low-signal-intensity area on T2-weighted images, representing the smooth muscle hyperplasia and the heterotopic endometrial tissue. T2-weighted sequences play a key role for MRI diagnosis of adenomyosis since they highlight the JZ, which has commonly increased thickness ^49^. Furthermore, on T2-weighted MRI, small high-signal-intensity areas refer to ectopic endometrium and also small intramyometrial cysts may be detected. However, T1-weighted sequences also contribute to the diagnosis as they are useful in identifying high-signal-intensity foci representing areas of hemorrhage ^49^. The detection of bright hemorrhagic foci has a high positive predictive value (95%) for the diagnosis of adenomyosis but a low sensitivity (47.5%) ^31^. The common appearance of adenomyosis is an enlarged, asymmetric uterus, where adenomyotic tissue is located mainly in the posterior wall or at the fundus. The most frequent finding to diagnose adenomyosis is the thickening of JZ, and several criteria have been proposed (JZ of at least 8–12 mm, the maximum JZ/total myometrium ratio of over 40%, and a difference between the maximum and the minimum thickness of greater than 5 mm); however, a thickness exceeding 12 mm seems to be highly predictive of adenomyosis ^31, 50^. Nevertheless, results are controversial as JZ may change on the basis of hormonal status (postmenopausal condition and use of hormonal contraception) and phase of menstrual cycle ^51^. During menstruation, the uterus may present with a marked thickening of the JZ, mimicking adenomyosis; thus, MRI evaluations should preferably be performed in the late proliferative phase. Furthermore, a common pitfall to consider is caused by transient uterine contractions that can mimic either T2-weighted hypointense bands perpendicular to the JZ or focal thickening of the JZ ^52^. In those cases, the repetition of MRI acquisition may help to differentiate a physiological condition from adenomyosis ^53^.

A JZ of less than 8 mm generally allows clinicians to exclude the presence of adenomyosis ^42^, whereas a measurement between 8 and 12 mm identifies the condition of adenomyosis if other criteria are present, such as maximal JZ thickness–to–myometrium thickness ratio over 40% or a relative thickening of the JZ in a localized area ^54^. Another criterion to be considered is a difference between the maximum and the minimum thickness of the JZ measuring more than 5 mm ^54^. However, adenomyosis may also be identified in the case of a poorly defined JZ or in the presence of linear striations of high T2 signal radiating from the endometrial zona basalis into the myometrium.

According to the most recent review on imaging of adenomyosis, MRI has a pooled sensitivity of 0.77, specificity of 0.89, positive likelihood ratio of 6.5, and negative likelihood ratio of 0.2 for all subtypes, showing better results compared with those of TVUS ^39^. However, MRI should be considered a second-line imaging technique and should be performed by expert radiologists.

## Coexistence with other gynecological conditions

Adenomyosis frequently coexists with other gynecological diseases, such as endometriosis ^25^ and uterine fibroids ^55, 56^. In 15% to 57% of cases, uterine leiomyomas and adenomyosis coexist in the same uterus and women with both conditions are more likely to experience pelvic pain ^57^. Results from a case-control study on women undergoing hysterectomy showed that women with leiomyoma and adenomyosis were more likely to report dysmenorrhea, dyspareunia, and non-cyclic pelvic pain compared with women with leiomyomas only ^3, 57, 58^.

Adenomyosis and endometriosis share a number of features, so that for many years adenomyosis has been called *endometriosis interna*. Nevertheless, they are considered two different entities because of specific pathogenic pathways and clinical presentation, although they often coexist in the same patients. Controversial results are available from a surgical dataset showing that adenomyosis prevalence in women with endometriosis ranges from 20% to 80% ^25, 26^. Besides, on ultrasound pre-operative assessment, 47.8% of patients undergoing surgery for DIE were affected by adenomyosis, and in those affected by both conditions, the surgical treatment was not as effective in treating pain as it was in those with only endometriosis ^59^. Furthermore, DIE phenotype was also shown to be associated with a specific form of focal adenomyosis located in the outer myometrium ^17^. However, larger samples of pre-operative cases are needed in order to better estimate the prevalence of gynecological comorbidities.

## Impact on fertility

Adenomyosis has been considered the typical uterine condition of multiparous women, although an increasing amount of evidence suggests an association with infertility and reproductive failure ^60– 63^. In a recent cross-sectional study on infertile women, adenomyosis prevalence were 24.4% in women at least 40 years old and 22% in women less than 40 years old. This percentage increased to 38.2% in cases of recurrent pregnancy loss and to 34.7% in previous ART failure ^21^.

Currently, infertility is considered one of the possible clinical presentations of adenomyosis and several theories have been proposed to explain the underlying mechanisms ^7^. Abnormal utero-tubal transport seems to be an important mechanism leading to infertility and is due to anatomical distortion of the uterine cavity but also to disturbed uterine peristalsis and sperm transport ^64^. The inner myometrium and, in particular, the JZ present with dysfunctional hyperperistalsis and increased intrauterine pressure. In addition, in the presence of adenomyosis, ultrastructural myometrial abnormalities cause a disturbance in normal myocyte contractility with subsequent loss of normal rhythmic contraction ^65^.

In infertile women with adenomyosis, eutopic endometrium shows a wide variety of molecular alterations, causing an altered receptivity ^13, 66^. This includes altered sex steroid hormone pathway, increased inflammatory markers and oxidative stress, reduced expression of implantation markers, lack of expression of adhesion molecules, and altered function of the gene for embryonic development (HOXA 10 gene), causing an impairment of implantation in women with adenomyosis ^7^.

There are no available studies on natural conception in adenomyosis, but several papers evaluated the effect of adenomyosis in women undergoing ART or in those surgically treated for DIE ^67^, and results are controversial. The meta-analysis published by Vercellini *et al*. in 2014 ^60^ reported rates of miscarriages of 31% in women with adenomyosis and 14.1% in non-affected women (relative risk (RR) 2.12, 95% confidence interval (CI) 1.20–3.75) ^60^. On the contrary, a case-control study in a group of women undergoing *in vitro* fertilization (IVF) showed that implantation rate was not significantly impaired in those diagnosed with adenomyosis at TVUS, but asymptomatic for AUB, compared to those not affected by the disease ^68^. On the contrary, Mavrelos *et al*. ^69^, in a multicenter prospective study, showed that the estimated probability of clinical pregnancy decreased from 42.7% in women with no adenomyosis to 22.9% in those with four and 13.0% in those with all seven ultrasound features of adenomyosis. This suggests that the severity of the condition, expressed as a number of morphological features on ultrasound, worsens the reproductive outcome. Results from the most recent systematic review and meta-analysis on IVF treatment outcomes in adenomyosis, including 11 studies and 519 patients with TVUS or MRI diagnosis of adenomyosis, confirmed the detrimental effect of the uterine disease on reproductive outcome ^61^. Rates of implantation, clinical pregnancy per cycle, clinical pregnancy per embryo transfer, ongoing pregnancy, and live-birth rate among women with adenomyosis were significantly reduced, whereas miscarriage rate was increased. The study also demonstrated that a pre-IVF treatment with the use of gonadotropin-releasing hormone analogue (GnRHa) down-regulation may be beneficial to the pregnancy rate ^61^.

Concerning the association between endometriosis and adenomyosis and fertility, results from a systematic review and meta-analysis published in 2014 on women after surgery for rectovaginal and colorectal endometriosis reported a 68% reduction in the likelihood of pregnancy in women who wanted to become pregnant ^70^. In a recent retrospective cohort of women undergoing IVF, the presence of adenomyosis affected clinical pregnancy rate, live-birth rate and miscarriage rate. In particular, compared with women with endometriosis alone, those affected by adenomyosis had a significantly lower clinical pregnancy rate (26.4% versus 12.5%) and live-birth rate (26.4% versus 12.5%). Similar results were reported in women with endometriosis and adenomyosis ^71^.

All of this evidence supported a negative effect of adenomyosis on reproductive outcome; however, it is essential to define strict criteria for imaging diagnosis and classification of adenomyosis in order to design and then compare homogeneous studies. This would allow clinicians to evaluate whether the severity and extent of disease have an additional negative effect and determine the therapeutic options to improve fertility in patients with adenomyosis.

## Impact on pregnancy outcome

Adenomyosis is considered a reproductive disorder and an increasing number of papers are showing that not only fertility but also pregnancy outcome is affected. Given late pregnancy outcomes, an increased risk of preterm birth (PTB) (adjusted odds ratio [aOR] 1.84, 95% CI 1.32–4.31) and preterm premature rupture of membranes (aOR 1.98, 95% CI 1.39–3.15) in patients with adenomyosis was shown ^72^. These results were confirmed in a small cohort of women diagnosed by ultrasound or MRI before pregnancy, showing also a significantly higher risk of cesarean delivery (OR 4.5, 95% CI 2.1–9.7), small for gestational age (SGA) fetuses (OR 4.3, 95% CI 1.8–10.3), postpartum hemorrhage (OR 6.5, 95% CI 2.2–19.0), and fetal malpresentation (OR 4.2, 95% CI 1.6–10.8) ^73^. Furthermore, a recent retrospective case-control study reported that adenomyosis is also associated with increased risk of second-trimester miscarriage (OR 11.2, 95% CI 2.2–71.2), pre-eclampsia (OR 21.0, 95% CI 4.8–124.5), and placental malposition (OR 4.9, 95% CI 1.4–16.3) ^74^. The type of adenomyosis may influence the pregnancy outcome, as shown by the higher rates of pregnancy-induced hypertension and uterine infection in patients with diffuse-type adenomyosis than in patients with focal-type adenomyosis. In addition, as found in the same study, the rates of cervical incompetency increased according to the extent of adenomyosis ^75^. Very recently, a prospective Japanese nationwide birth cohort study was published, and according to results obtained from self-reported questionnaires, adenomyosis was a risk factor for PTB of less than 37 weeks (aOR 2.49, 95% CI 1.89–3.41), PTB of less than 34 weeks (aOR 1.91, 95% CI 1.02–3.55), low birth weight of less than 2500 g (aOR 1.83, 95% CI 1.36–2.45), low birth weight of less than 1500 g (aOR 2.39, 95% CI 1.20–4.77), and SGA neonates (aOR 1.68, 95% CI 1.13–2.51) ^76^.

Concerning the pathogenic mechanisms involved in obstetric complications in adenomyosis, the role of inflammation, increased myometrial prostaglandin production, altered uterine contractility, and intrauterine pressure was hypothesized to explain the link with PTB ^77^. In adenomyosis, an activation of local and systemic inflammatory pathways was shown, influencing the decidua–trophoblast interactions early in gestation as well as chorion–decidua interactions that could activate mechanisms of PTB later in pregnancy ^77^. In regard to the increased rate of placenta-related disorders, the defective myometrial spiral artery remodeling and deep placentation can be considered to be among the major causes of obstetrical syndromes in adenomyosis. In fact, alterations in the inner myometrium and in the uterine JZ have been well documented ^12, 78^. However, further research protocols are needed from both the epidemiological and physio-pathological point of view. In fact, well-conducted studies may be performed only if uniform and shared imaging diagnostic criteria and classification are used to make the pre-pregnancy diagnosis of adenomyosis. Similarly, the lack of evidence on molecular mechanisms leading to obstetric complications in adenomyosis should be filled.

## Management of adenomyosis

Adenomyosis has a negative impact on women’s quality of life in a high percentage of cases because of AUB and pain requiring a lifelong management plan through medical or surgical treatment ^79^. The choice depends on the woman’s age, reproductive status, and clinical symptoms. However, so far, few clinical studies focusing on medical or surgical treatment for adenomyosis have been performed, and no drugs labelled for adenomyosis are currently available ^8^. Nonetheless, the disease is increasingly diagnosed in young women with reproductive desire, and conservative treatments should be preferred.

The surgical approach remains a controversial subject ^80^, but minimally invasive surgical treatments should be performed in specific cases, informing the patient about the potential risks in case of pregnancy. Conservative surgical options include endometrial ablation, hysteroscopic endometrial and adenomyoma resection, laparoscopic resection of adenomyosis, high-intensity focused ultrasonography, and uterine artery embolization ^81^. However, robust evidence supporting conservative surgical treatments of adenomyosis is still lacking.

According to pathogenic mechanisms, several medical hormonal and non-hormonal treatments are used off-label to manage pain and bleeding and to improve fertility outcome. The use of GnRHa is indicated before fertility treatments to improve the chances of pregnancy in infertile women with adenomyosis ^61^, and the highest pregnancy rate is reported in those undergoing frozen embryo transfer after GnRHa pre-treatment ^82^. In contrast, the use of GnRHa for pain and bleeding should be considered only for short-term treatment because of menopausal effects.

The use of progestins is supported by the anti-proliferative and anti-inflammatory effect and decidualization and then atrophy of endometrial tissue, causing a significant reduction in bleeding ^8^. Among progestins, norethisterone acetate (NETA), vaginal danazol, and dienogest (DNG) may be considered. Recently, a randomized, double-blind, multicenter, placebo-controlled trial on DNG, daily administered for 16 weeks in women with adenomyosis, showed a significant decrease of pain score in those treated ^83^. The results were confirmed in a long-term treatment study, demonstrating good tolerability and a reduction in pain and higher quality-of-life scores ^84^. The levonorgestrel-releasing intrauterine system (LNG-IUS) is also an effective, reversible, and long-term treatment used successfully to treat adenomyosis. Results show that it reduces menstrual bleeding, pain, and uterine volume and has an overall satisfaction of 72% ^85^. However, new drugs, such as selective progesterone receptor modulators, aromatase inhibitors, valproic acid, and anti-platelet therapy, are under development for the treatment of adenomyosis ^8^.

## Conclusions

In the last decade, a significant improvement has been achieved in understanding and management of adenomyosis. Adenomyosis has become a clinical entity rather than just a histological diagnosis and it can be identified through non-invasive imaging techniques. An increasing amount of evidence is showing the pathogenic mechanisms involved and the potential medical treatments. However, there is still the urgent need for a uniform and shared diagnostic criteria profile and reporting system, in both imaging and histology, in order to identify all of the clinical and imaging phenotypes of adenomyosis. This is the first step to share a common language among scientists and clinicians, who would share the same diagnostic criteria. This would allow clinicians to design and perform methodologically well-conducted prospective studies on adenomyosis prevalence, gynecological comorbidities, effectiveness of medical or surgical treatments, and impact on fertility and pregnancy outcome.

## Abbreviations

aOR, adjusted odds ratio; ART, assisted reproductive technology; AUB, abnormal uterine bleeding; CI, confidence interval; DIE, deep infiltrating endometriosis; DNG, dienogest; FOAM, focal adenomyosis of the outer myometrium; GnRHa, gonadotropin-releasing hormone analogues; HMB, heavy menstrual bleeding; IVF, *in vitro* fertilization; JZ, junctional zone; MRI, magnetic resonance imaging; OR, odds ratio; PTB, preterm birth; RR, relative risk; SGA, small for gestational age; TIAR, tissue injury and repair; TVUS, transvaginal ultrasonography.
